# Effect of the chromaticity of stimuli on night vision disturbances

**DOI:** 10.1038/s41598-024-61069-4

**Published:** 2024-05-03

**Authors:** José J. Castro-Torres, Miriam Casares-López, Sonia Ortiz-Peregrina, Francesco Martino, Luis Gómez-Robledo, José R. Jiménez

**Affiliations:** 1 Laboratory of Vision Sciences and Applications, Department of Optics, University of Granada, 18071 Granada, Spain; 2 Basic and Applied Colorimetry Lab, Department of Optics, University of Granada, 18071 Granada, Spain

**Keywords:** Health care, Risk factors, Optics and photonics

## Abstract

The perception of halos and other night vision disturbances is a common complaint in clinical practice. Such visual disturbances must be assessed in order to fully characterize each patient’s visual performance, which is particularly relevant when carrying out a range of daily tasks. Visual problems are usually assessed using achromatic stimuli, yet the stimuli encountered in daily life have very different chromaticities. Hence, it is important to assess the effect of the chromaticity of visual stimuli on night vision disturbances. The aim of this work is to study the influence of the chromaticity of different visual stimuli on night vision disturbances by analyzing straylight and visual discrimination under low-light conditions. For that, we assessed the monocular and binocular visual discrimination of 27 subjects under low illumination using the Halo test. The subjects’ visual discrimination was assessed after exposure to different visual stimuli: achromatic, red, green, and blue, both at the monitor’s maximum luminance and maintaining the same luminance value for the different visual stimuli. Monocular straylight was also measured for an achromatic, red, green, and blue stimuli. The blue stimulus had the greatest effect on halos in both monocular and binocular conditions. Visual discrimination was similar for the red, green, and achromatic stimuli, but worsened at lower luminance. The greatest influence of straylight was observed for the blue stimulus. In addition, visual discrimination correlated with straylight measurements for achromatic stimuli, wherein greater straylight values correlated with an increased perception of halos and other visual disturbances.

## Introduction

The parameter most widely used in clinical practice to characterize visual function is visual acuity (VA) as it can be used, together with the visual field, to classify visual impairment^1^. Visual acuity also fulfills an important clinical application because it is used as the main metric to assess vision after refractive surgery^2^. Besides VA, interest has been growing in other visual functions, such as contrast sensitivity and stereopsis, which are not only important in the assessment of visual performance, but also when performing various daily tasks, such as reading or fine motor skills^3,4^. All these visual functions are usually measured under photopic conditions. However, under low illumination, the pupils dilate; consequently, halos and other night vision disturbances^5^ are more readily perceived by subjects exposed to intense light sources^6,7^. Even subjects with healthy eyes may also be affected by certain night vision disturbances in the presence of intense light sources^8,9^, including halos, because they are not diffraction-limited eyes and as the pupils dilate, they are affected by ocular aberrations and intraocular scattering^9,10^. In clinical practice, one of the main complaints of patients who have undergone refractive surgery are night vision disturbances (halos, glare, and starbursts), as changes to the ocular media can increase the amount of ocular aberrations, especially when the pupil is enlarged in low light conditions^11,12^. Furthermore, these visual symptoms can also be observed in some eye diseases^13–15^. Several studies indicate the importance of studying visual disturbances for a more complete characterization of visual performance^11,16^ or even in the diagnosis of certain eye diseases^17^. Although these positive dysphotopsias, i.e. photic phenomena or introduction of bright artifacts onto the retina^18^, are often assessed through questionnaires^12^, increasingly more tests are being developed to characterize them^15,19–21^. These tests for night vision disturbances have been used to assess normal vision^10,19^ and some clinical conditions^15,17,22^. Thus, as cataract patients often complain about postoperative halo perception, night vision tests can be used to analyze the outcomes of implanting different IOLs (intraocular lenses)^22,23^. Night vision disturbances following LASIK (Laser In Situ Keratomileusis) surgery have also been studied and were found to deteriorate under several experimental conditions^24,25^. With other techniques such as small incision lenticule extraction (SMILE), halo symptoms appeared early after surgery, but improved within 3 months^26^, showing the importance of studying the evolution of night vision disturbances after refractive surgery. Pupil size is another factor to consider, as pupil dilation exacerbates ocular aberrations and intraocular scattering^27,28^, which may affect visual function and contribute to increased halo perception^6^ and other dysphotopsias^29^. Pupil size is also an important parameter following corneal refractive surgery, given that patients with larger pupils report higher rates of night vision disturbances. Furthermore, these visual symptoms had a negative effect on certain tasks, such as night driving^30^. The tests used to assess night vision disturbances are mostly designed and performed with achromatic stimuli. However, different chromatic stimuli, e.g., streetlights, illuminated signs, headlights, taillights, etc., are regularly encountered during everyday nighttime activities. Several studies have also shown that intraocular scattering and straylight depend on the wavelength^31,32^. On the other hand, it has been reported that retinal image quality affects night vision performance^10^. Considering these findings, we believe it is important to assess visual performance in dim environments using not only achromatic stimuli, but also with stimuli of different chromaticities.

The aim of this work is to assess the influence of the chromaticity of visual stimuli on night vision disturbances and intraocular straylight. To that end, we measured and compared visual discrimination and intraocular straylight under low illumination (halo perception) in a group of subjects with healthy eyes using achromatic and chromatic luminous stimuli (red, green, and blue) and by analyzing the effects of chromaticity in different situations.

## Methods

### Subjects

A total of 27 subjects (16 females, 11 males) with a mean age of 22.9 ± 4.0 years were enrolled in the study. All participants gave their informed consent in accordance with the Declaration of Helsinki, and the study was prospectively approved by the Human Research Ethics Committee of the University of Granada (1256/CEIH/2020). The subject inclusion criteria were decimal best-corrected visual acuity ≥ 1.0 in both eyes and no pathological conditions or pharmacological treatments that could affect visual performance. Subjective refraction was performed in both eyes at distance using an endpoint of maximum plus for best visual acuity. The assessment of monocular refraction was followed by a binocular balance using the prism-dissociated red-green balance technique^33^. Corrected distance visual acuity (CDVA) was measured at a working distance of 5.5 m using the Pola VistaVision Visual Acuity Chart System (DMD Med Tech, Torino, Italy). The mean refractive error (spherical equivalent) was − 1.00 ± 1.60 D. All observers completed the Ishihara test (Kanehara Shuppen Company, Ltd., Tokyo, Japan) and none had any color vision deficiency.

### Visual discrimination (halo perception)

Visual discrimination is referred to in the present study as the ability of the visual system to detect luminous stimuli around a main high-luminance stimulus over a dark background^15^. We assessed halo perception (i.e., the subject’s perception of an area of diffused light around an intense light) and other night vision disturbances by studying visual discrimination under low-light conditions (in a dark room). Halos and positive dysphotopsias have been extensively studied in clinical applications and are mainly caused by forward scattering^34^, although defocus and ocular aberrations are also involved^19,35^. Here we used the Halo test to analyze these visual disturbances. The test is based on the freely available software Halo v1.0 (http://hdl.handle.net/10481/5478, University of Granada, Granada, Spain), and has been extensively validated in different clinical applications, including for eye diseases^15,36^, and after refractive surgery^22,23,25^, but also under diverse experimental conditions, such as anisocoria^6^, after inducing forward scattering^10,37^ or under the influence of diverse substances^38,39^. The visual task consists of detecting peripheral luminous stimuli around a central high-luminance stimulus. To perform the test, the subjects were positioned 2.5 m in front of the test monitor with their visual axis aligned with the center of the monitor using a chin rest and a forehead support. Subjects were instructed to maintain visual fixation on the central stimulus throughout the test. The task of the subject was to detect peripheral spots around the central stimulus by pressing the left mouse button of the test computer. An initial trial run was performed to check they understood the test correctly. The central and peripheral stimuli subtended visual angles of 0.390 and 0.013°, respectively. The assessment area comprised a ring concentric to the central stimulus limited by minimum and maximum radii of 0.195 and 0.390°, respectively, hence the test was performed under conditions of foveal vision. A total of 60 peripheral stimuli, distributed along 15 semiaxes (4 stimuli/semiaxis), were displayed around the central stimulus and within the area being assessed. The monitor’s background luminance was 0.27 cd/m^2^. Subjects were tested in a completely darkened room (mesopic conditions) after completing a dark adaptation of 3 min plus 1 min to adapt to the central stimulus luminance. The peripheral stimuli were randomly displayed in the assessment area after a randomly selected interval of between 0.8 and 2.5 s. Each peripheral stimulus was displayed once with a duration of 0.8 s at each position. Each time the subject perceived a peripheral stimulus (by pressing the mouse button), the response was stored for subsequent analysis and calculation of the corresponding index. The metric obtained is the visual disturbance index (VDI), which is calculated from the number of undetected stimuli versus the total number of luminous stimuli (weighted by the square of the distance between each peripheral stimulus and the center of the main stimulus)^15^ using the Eq. (1):

1 $$VDI=\left(\sum \limits_{i=1}^{N}{p}_{i}{r}_{i}^{2}\right)/\left(\sum \limits_{i=1}^{N}{r}_{i}^{2}\right),$$

where r_i_ is the distance (in pixels) from the center of the central stimulus to the center of the i-peripheral stimulus, for a concrete semi-axis; N is the total number of peripheral stimuli (N = 60); and p_i_ take values of 0 or 1 if the i-stimulus is detected or non-detected by the subject, respectively. The VDI takes values from 0 (all peripheral stimuli detected) to 1 (none detected) and, therefore, a higher VDI is indicative of poorer visual discrimination and a greater influence of halos and other dysphotopsias. The halo test also provides a results graph showing, for each of the positions in which a peripheral stimulus was displayed, whether the participant detected it or not, using the character 1 in green or the character X in red, respectively.

**Figure 1:**
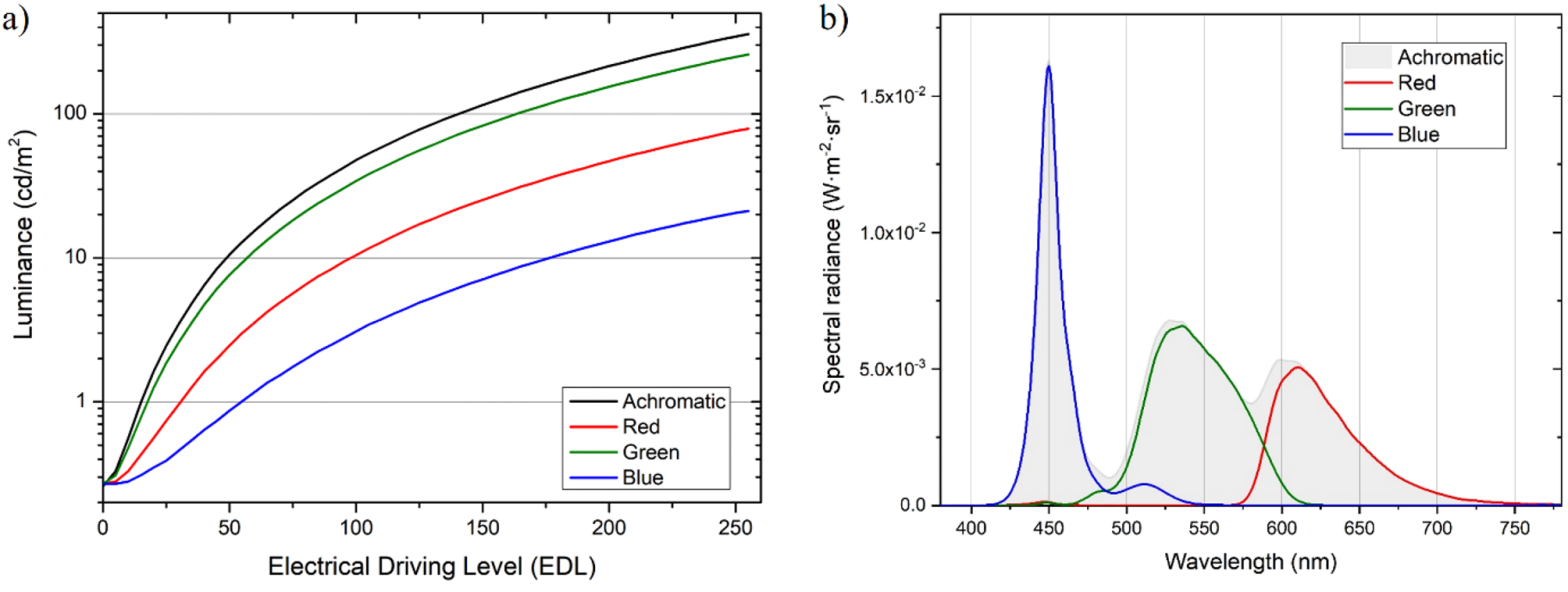
(**a**) Electro-optical transfer functions (EOTFs) of the display used in the Halo test for the red, green, and blue channels and an achromatic state. (**b**) Spectral radiance for achromatic and chromatic stimuli (R, G, B) at maximum EDL values.

The visual stimuli were presented on an LCD monitor (Benq Mobiuz EX2510 IPS FHD, 24.5 inches), which was characterized using the spectroradiometer CS-2000 (Konica Minolta, Inc. Osaka, Japan), in which a field of 1° was previously selected. Luminance measurements were made at the center of the monitor by varying the electrical driving level (EDL), from the maximum value (EDL of 255) to the dark state (EDL of 0) in steps of 5-EDL-units displaying on the monitor the visual stimulus used in the Halo test. As a result of these measures, Fig. 1a shows the electro-optical transfer functions (EOTFs) of the monitor for the achromatic (A) state and for the red (R), green (G), and blue (B) channels^40^. Figure 1b shows the spectral radiance for achromatic and chromatic stimuli (R, G, B) for maximum EDL values.

Considering the experimental conditions used in previous studies, the Halo test uses achromatic stimuli, i.e., stimuli devoid of tone (grayscale), in such a way that the main stimulus shows the highest luminance, with EDL values for R, G, B all equal to 255, and the peripheral stimuli emitting less luminance. In the present study, we performed the Halo test using an achromatic stimulus and three chromatic stimuli (red, blue, and green) for two luminance conditions: (1) maximum luminance for each chromatic stimuli (an EDL of 255 for the corresponding channel of the main stimulus), and (2) constant luminance for the different stimuli (Table 1). The blue channel was considered for the latter scenario, since it achieved the lowest luminance for each EDL. EDL values that provided the same blue channel luminance were interpolated for the red and green channels using the EOTFs (Fig. 1a). In all cases, the peripheral stimulus had the same luminance reduction (about 40%) as the central stimulus.

**Table 1:** Color configuration (in terms of EDL values and chromaticity coordinates, CC) and luminance (cd/m^2^) of the main and peripheral stimuli for the different experimental conditions: maximum luminance, Lmax, and maximum luminance of the blue channel, L_Bmax. A: achromatic; R: red; G: green; B: blue.

Luminance condition		Main stimulus		Peripheral stimulus	
		EDL (R, G, B) CC (x, y)	L (cd/m^2^)	EDL (R, G, B) CC (x, y)	L (cd/m^2^)
Lmax	Amax	(255, 255, 255) (0.3120, 0.3227)	358.2	(198, 198, 198) (0.3120, 0.3227)	210.2
	Rmax	(255, 0, 0) (0.6455, 0.3404)	79.1	(199, 0, 0) (0.6447, 0.3403)	46.5
	Gmax	(0, 255, 0) (0.3095, 0.6398)	259.2	(0, 198, 0) (0.3104, 0.6389)	151.4
	Bmax	(0, 0, 255) (0.1508, 0.0443)	21.2	(0, 0, 196) (0.1508, 0.0455)	12.4
L_Bmax	A_Bmax	(68, 68, 68) (0.3108, 0.3213)	21.2	(54, 54, 54) (0.3100, 0.3200)	12.4
	R_Bmax	(138, 0, 0) (0.6410, 0.3395)	21.2	(108, 0, 0) (0.6364, 0.3383)	12.4
	G_Bmax	(0, 80, 0) (0.3088, 0.6282)	21.2	(0, 63, 0) (0.3076, 0.6197)	12.4

Pupil size under the experimental conditions of the Halo test was measured using a Colvard pupillometer (OASIS Medical, Inc., Glendora, USA). For conditions in which the luminance of the central stimulus was the same, only the pupil size for the blue central stimulus was measured. Therefore, pupil size was measured at maximum luminance for each stimulus, i.e., under Lmax conditions. We also measured the illuminance at the observer’s position under the Halo test conditions using a PCE-170A light meter (PCE Instruments, PCE Deutschland GmbH, Germany). The Halo test display was the only light source in the room (dim environment) and displayed the corresponding central stimulus (Halo test running). An illuminance of 0.01 lx was measured for the achromatic stimulus when under conditions of maximum luminance, which coincides with the sensitivity of the instrument. The device did not register any values for the other conditions (0.00 lx).

### Intraocular straylight

**Figure 2:**
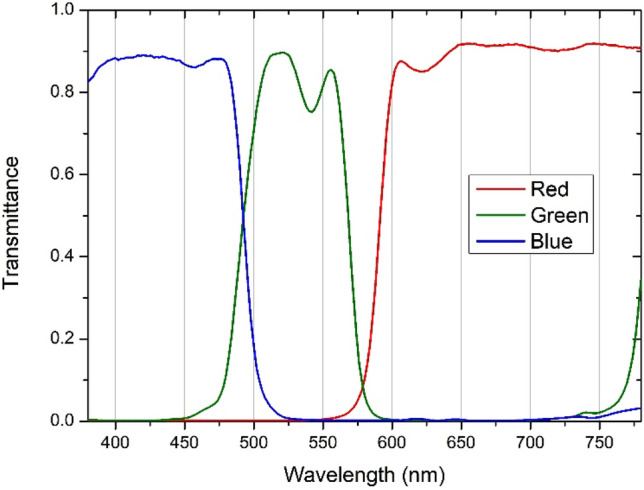
Spectral transmittance for the colored filters used in the straylight measurements: red, green, and blue.

Intraocular straylight was measured using a commercial straylight meter (C-Quant, Oculus GmbH, Wetzlar, Germany). The device employs the psychophysical compensation comparison method and has been extensively validated for clinical applications^41–44^. The meter returns results in terms of the straylight parameter [log(s)], which is the ratio between the scattered and unscattered light reported by the subject. A high value of the log(s) indicates a higher amount of forward intraocular straylight and, therefore, a stronger luminous veil over the retinal image. According to the normal straylight formula^41^, log(s) takes values of about 0.90 for young healthy eyes, 1.03 at 50 years of age, and 1.42 for 80-year-old patients. The analysis only included values that met the reliability criterion, i.e., all values with an expected standard deviation (ESD) in each individual measurement of less than 0.08. This device has been shown to be repeatable and reliable for intraocular straylight assessment^45^. Straylight measurements were performed monocularly under natural viewing conditions (achromatic stimulus) and using three different additive dichroic filters (red, green, and blue), whose spectral transmittance is shown in Fig. 2. We also evaluated whether the filter material induced light scattering. For that, we used the OQAS II double-pass device (Visiometrics, Terrassa, Spain) which uses an infrared laser diode (780 nm) to obtain the double-pass image. We measured the objective scattering index (OSI) of an artificial eye, as made in other studies^10^, and interposing each of the chromatic filters. It was found that the OSI did not vary (OSI = 0.0), except for the blue filter, which could not be measured due to its low spectral transmittance above 750 nm.

### Procedures

The Halo test was performed monocularly in both eyes and binocularly following a random pattern and using different stimuli (achromatic, red, green, and blue at the maximum luminance of the central stimulus, i.e., for EDL values of 255; and achromatic, red, and green with a central stimulus luminance equal to the maximum luminance of the blue channel). During the test, the participants wore their best optical correction (obtained in subjective refraction). We completed one Halo test measurement for each experimental condition. The participants performed a preliminary trial in the first session to familiarize themselves with the visual test. Straylight measurements were performed monocularly under natural viewing conditions and those created by placing each of the colored filters (red, green, and blue) on the device’s eyepiece, whose spectral transmittance is shown in Fig. 2. The experiment was scheduled over different sessions to avoid subject fatigue.

### Statistical analysis

SPSS v.26 software (SPSS Inc., Chicago, IL) was used to analyze the raw data. The Shapiro–Wilk test was applied to check the normality of the data distribution for the measured parameters [VDI and log(s)]. To study how the two parameters were affected by each type of stimulus, we applied a repeated measures ANOVA test for normally distributed data and Friedman’s ANOVA test for non-normally distributed data. In both cases, we also carried out post-hoc pairwise comparisons with a Bonferroni correction. To determine the effect size, Hedges’g was calculated for non-normally distributed data. A correlation analysis was conducted to study the relationship between VDI and log(s) using the Pearson correlation test (r). Statistical significance was set at a p-value of 0.05 (p < 0.05).

## Results

**Figure 3:**
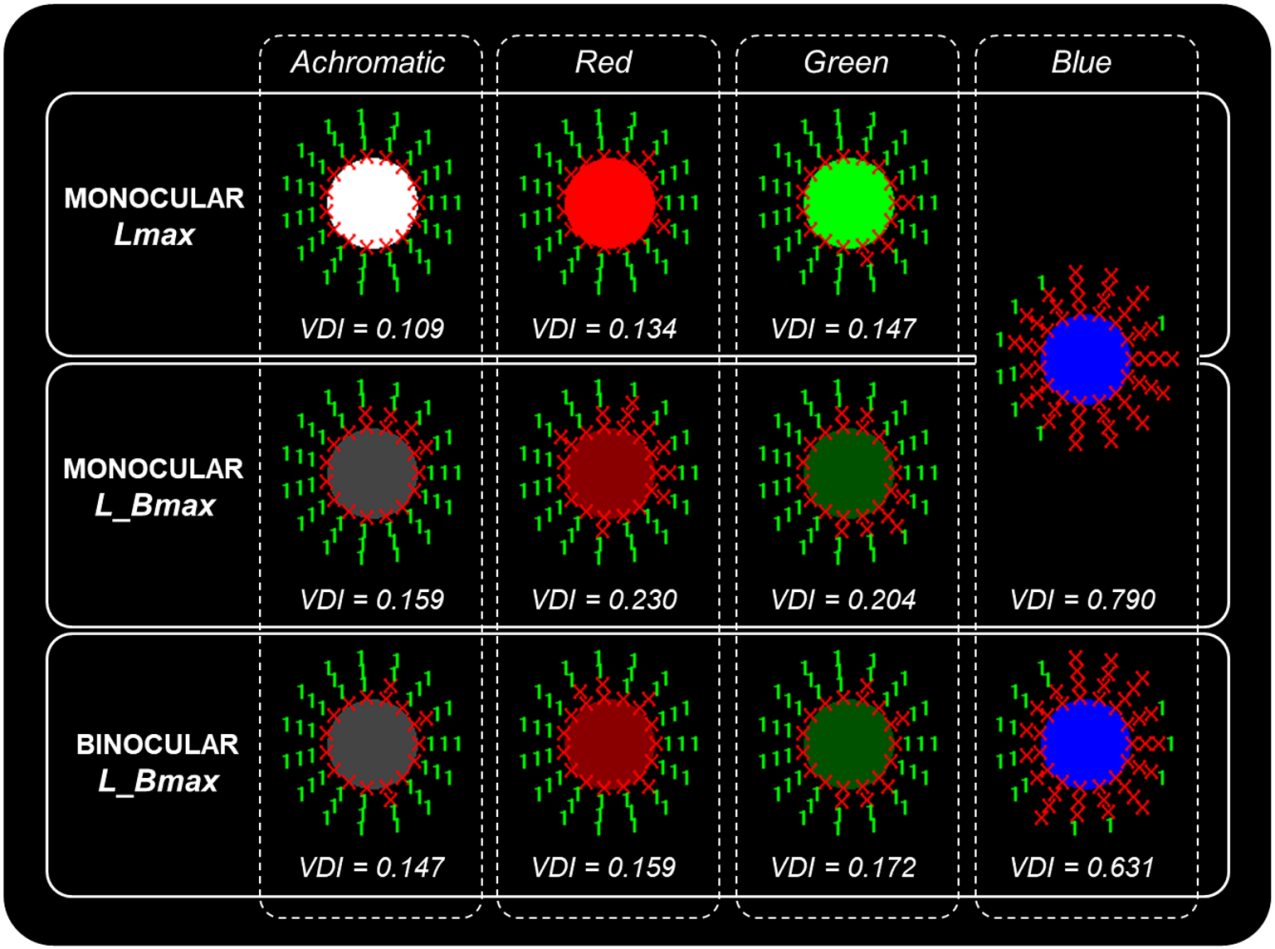
Graphical results of the Halo test and VDI values for one subject under the different conditions studied (achromatic/chromatic), monocularly (right eye) and binocularly. Red X: peripheral stimulus not detected; green number 1: peripheral stimulus detected.

Figure 3 shows the graphical results of the Halo test for a participant with a low halo perception under normal conditions (Amax), a result that is within the normal range^6^. However, as the luminance of the stimuli decreased, the halo perception increased, with the worst result obtained for the blue stimulus, where the halo covered almost the entire assessment area. Under binocular conditions, the perceived halo was smaller for all stimuli and luminance levels compared to monocular tests, i.e., visual discrimination capacity improved under natural viewing conditions (binocular vision). The figure also shows that some zones of the evaluated area with good visual discrimination maintained the same level under the different experimental conditions (see the zone on the left for each graphical result) and, therefore, these were also the areas where the subject had their best peripheral stimuli detection results.

**Figure 4:**
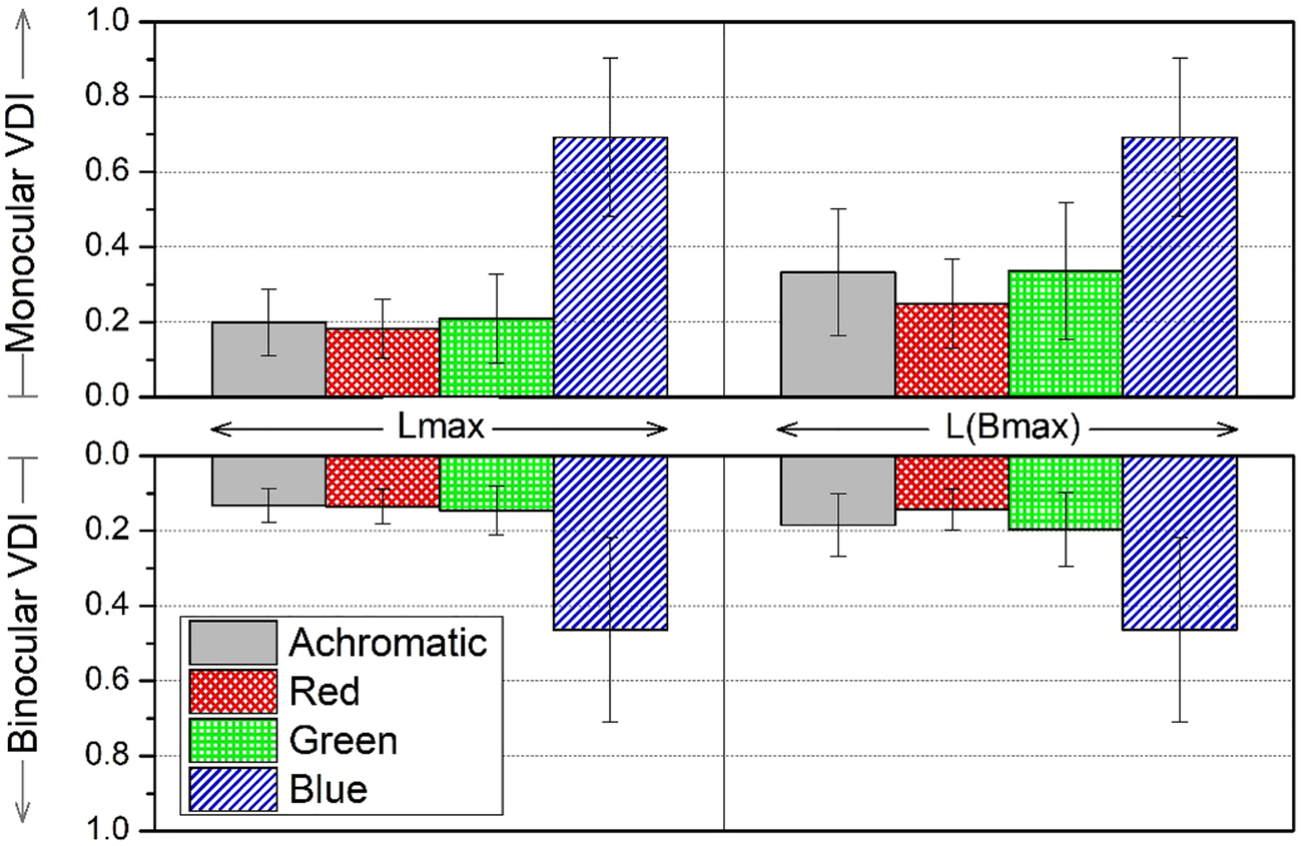
Bar graph showing the mean values of the monocular and binocular visual disturbance indices (VDI) under different conditions (achromatic, red, green, and blue) for the maximum luminance of the central stimulus, Lmax (EDL values of 255) and for the maximum luminance of the blue channel, L(Bmax). Standard deviations are shown by the vertical bars.

The visual discrimination results are shown in Fig. 4, which includes mean values of the monocular and binocular visual disturbance indices (VDI). Both luminance conditions are shown: Lmax and L_Bmax. Monocularly, we found significant differences in the VDI under both luminance conditions, Lmax (χ^2^(3) = 44.393; p < 0.001; *g* = 1.744) and L_Bmax (χ^2^(3) = 44.151; p < 0.001; *g* = 1.409). Pairwise comparisons showed that the VDI for the blue stimulus was significantly higher (p < 0.001) than that of the achromatic, red, and green stimuli in both luminance conditions, Lmax and L_Bmax. This indicates a poorer visual discrimination for the blue stimulus compared to the rest of the conditions. In addition, according to the Wilcoxon test, the monocular VDI was significantly higher at the lower luminance, L_Bmax, for the achromatic (Z = 3.772; p < 0.001; *g* = 0.788) and for the green chromatic condition (Z =  − 2.946; p = 0.003; *g* = 0.601) compared to the high-luminance condition, which indicates that halos have a stronger influence.

Binocularly, we found similar results. The Friedman test revealed significant differences in VDI between the two luminance conditions, Lmax (χ^2^(3) = 44.393; p < 0.001;* g* = 1.744) and L_Bmax (χ^2^(3) = 44.154; p < 0.001;* g* = 1.409), with pairwise comparisons showing a significantly higher VDI for the blue stimulus (p < 0.001) than that of the achromatic, red, and green stimuli. Comparisons of the different luminance conditions using the Wilcoxon test indicated significant differences in the binocular VDI for the achromatic state (Z =  − 3.772; p < 0.001;* g* = 0.788) and the green stimulus (Z =  − 2.946; p = 0.003; *g* = 0.601). The average VDI for the red stimulus, on the other hand, was higher under lower luminance conditions compared to Lmax condition (Fig. 4), although differences were not significant. Comparing the VDI of each monocular condition with its corresponding binocular VDI, the VDI was significantly lower in all cases for binocular viewing, which indicates a greater degree of visual discrimination. This underlines the superiority of the binocular system over monocular vision, as the visual discrimination was better in binocular viewing conditions.

Pupil size is another important parameter, considering the luminance of the stimuli changed during the Halo test. Table 2 shows the mean monocular VDI and pupil diameter (in mm) for the four different stimuli (achromatic/chromatic) under the two luminance conditions. For the L_Bmax condition, pupil size was only measured for the blue stimulus (equal luminance for the four stimuli) and we found that stimuli chromaticity had a significant effect (χ^2^(3) = 51.371; p < 0.001; *g* = 0.617). Pairwise comparisons of pupil diameter showed no significant differences between the achromatic, red, or green stimuli (p > 0.05). However, we obtained a significant increase in pupil diameter in response to the blue stimulus compared to the achromatic (p < 0.001), red (p = 0.002), and green (p = 0.001) ones.

**Table 2:** Mean monocular visual disturbance indices (VDI) and pupil sizes (in mm) under the different experimental conditions.

		Achromatic	Red	Green	Blue
Monocular VDI	Lmax	0.20 ± 0.09	0.18 ± 0.08	0.21 ± 0.12	0.69 ± 0.21
	L_Bmax	0.33 ± 0.17	0.25 ± 0.12	0.34 ± 0.19	
Pupil size (mm)	Lmax	5.2 ± 1.1	5.5 ± 0.8	5.5 ± 0.9	5.9 ± 1.0

**Figure 5:**
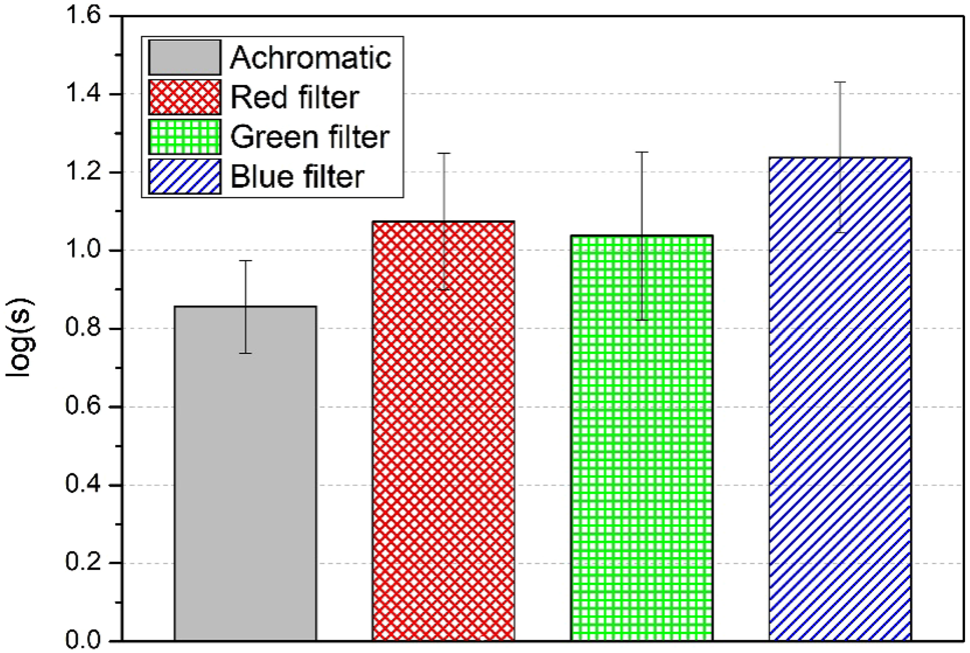
Mean log(s) for normal achromatic conditions and using different colored filters (red, green, and blue). Standard deviations are shown by the vertical bars.

With respect to the intraocular straylight measurements, Fig. 5 shows the mean log(s) under natural, achromatic conditions, as well as with each of the additive filters (red, green, and blue). We found that chromaticity had a significant effect on log(s) (χ^2^(3) = 120.277; p < 0.001; *g* = 0.883). Pairwise comparisons showed that log(s) was significantly lower for the achromatic stimulus compared to the three chromatic stimuli (p < 0.001), and no significant differences were found between the red and green filters (p = 0.340). Furthermore, log(s) while wearing the blue filter was significantly higher than that of the achromatic, red, and green stimuli (p < 0.001), which indicates the blue filter produces a greater degree of intraocular straylight.

**Figure 6:**
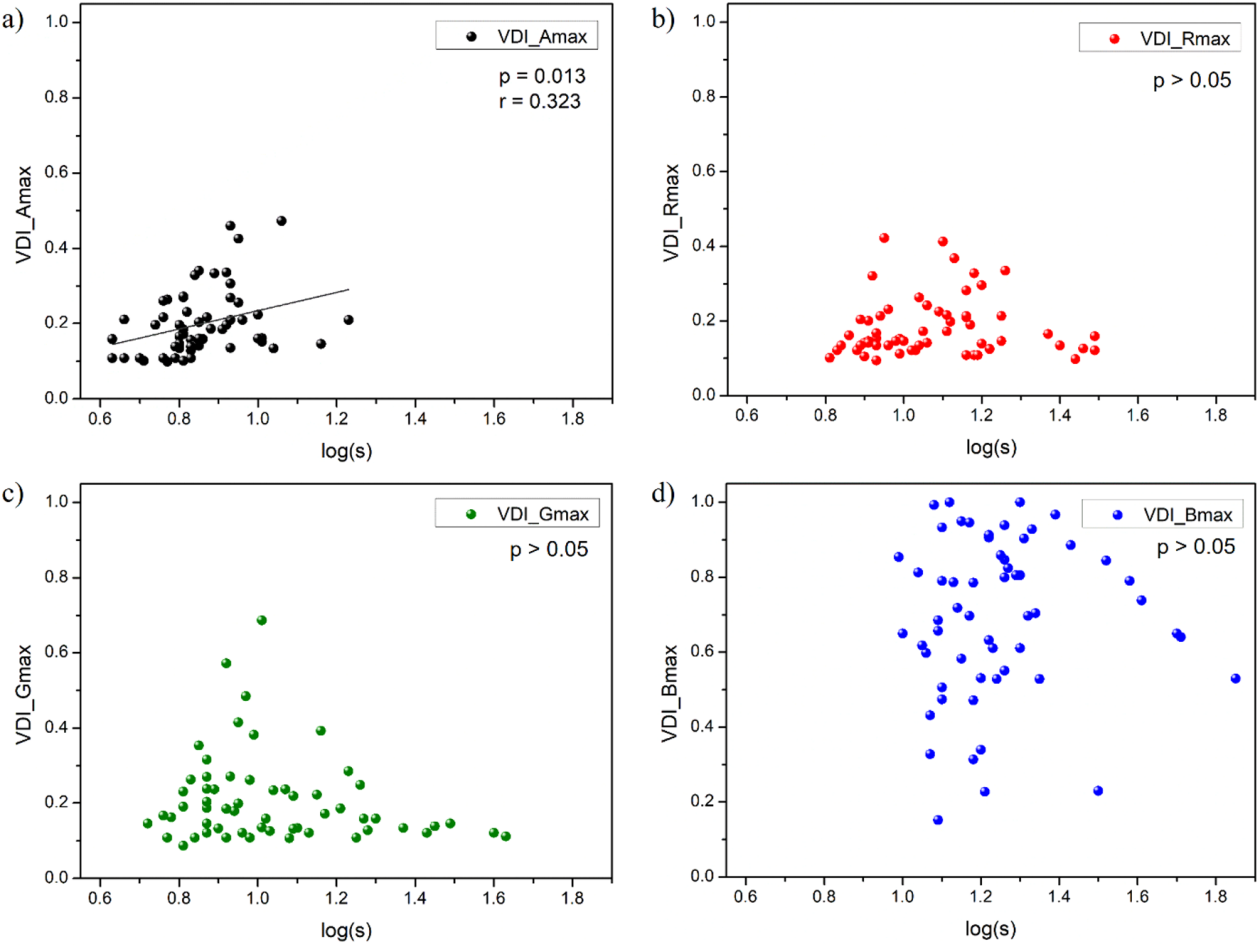
The monocular visual disturbance index (VDI) for maximum EDL as a function of intraocular straylight, log(s) under achromatic conditions (**a**) and for three chromatic settings: red (**b**), green (**c**), and blue (**d**).

Considering the achromatic and three chromatic conditions separately (Fig. 6), we found that there was only a significant, direct correlation between the monocular VDI and log(s) for the achromatic condition (p = 0.013; r = 0.323) (Fig. 6a), which means the subject’s visual discrimination declined more when their eyes showed a stronger effect of straylight.

## Discussion

The subjects’ visual discrimination was worse for the blue stimulus under both monocular and binocular testing. This impairment increased as the luminance of the stimuli was reduced, as a larger pupil diameter deteriorates the optical quality of the eye^27^. In addition, the visual disturbance index was lower under binocular viewing conditions for each of the experimental conditions. Other studies have reported that this binocular visual function is better than the monocular equivalent under different experimental conditions with achromatic stimuli, observing a positive binocular summation for the function^6^, which is in line with our results for different chromatic stimuli.

The increase in intraocular straylight observed with the blue filter agrees with the results of Coppens et al. who found a strong dependence of straylight on wavelength^31^. These authors suggested that straylight has three important components: a predominant, base component that follows the Rayleigh scattering pattern (greater degree of straylight at shorter wavelengths); a second component that depends on pigmentation, where less than perfectly pigmented eyes experience greater straylight at longer wavelengths; and a third, age-related component which means that intraocular straylight increases with age at all wavelengths. Our study population comprised young adults within a small age range, so we did not expect to find any significant age-related differences^41^. Furthermore, all participants were Caucasian, although in general they cannot be considered perfectly pigmented, so straylight might increase at longer wavelengths. In this regard, we generally observed that subjects experienced more straylight through the red filter, although the differences were not statistically significant compared to straylight produced by the green filter, which is in line with Coppens et al.^31^. One contribution to the increased straylight with the blue filter could be the Rayleigh pattern due to forward scattering. Although the C-Quant straylight meter is known to have good repeatability^45^, the differences in straylight between the green and red stimuli should be studied in more detail by increasing the number of measurements for each stimulus because they were close to the criterion reliability value and to confirm, therefore, that there was a slight increase in straylight for red stimuli. C-Quant measurements depends on subjective factors, since it uses a psychophysical method, and this aspect could have influenced a greater variability of the straylight especially for the blue condition. However, some authors have corroborated a good repeatability and reproducibility of the C-Quant in healthy eyes, but also a significant correlation between the straylight parameter and an objective scatter index (OSI) measured objectively using a double-pass device^46^, both parameters being good for clinical applications, as quantifying cataracts^47^. It is also observed that for the achromatic condition the straylight is lower than for the chromatic filters, a trend that is also observed in the Halo test. That is, both straylight and VDI are increased for chromatic stimuli. This may be due to a higher spectral sensitivity in the retina produced by the L, M and S cones^48^ when white light is used. For each chromatic condition (R, G or B), the sensitivity would be mainly limited by the spectral radiance of the luminous stimulus.

Other authors have used the double-pass technique to analyze the chromatic dependence of straylight at different angles, demonstrating that small angle straylight is affected by the wavelength-dependent properties of the fundus for wavelengths between 500 and 650 nm^32^. The same study also found a significant contribution from straylight at longer wavelengths because of light diffusing at the fundus, which depends on the subject’s pigmentation and could have a considerable impact in poorly pigmented eyes^32^. For wide angles, they found that the point spread function (PSF) and intraocular straylight had a weak dependence on wavelength. The authors also observed a minimum in straylight at approximately 550 nm, which is in line with our present findings. These results can be explained by the fact that the spectral sensitivity curve reaches a maximum at around the same wavelength, but they could be also caused by the combined effect of increased straylight for short (Rayleigh pattern for forward scattering) and long wavelengths (due to light diffusing at the fundus). These authors did not provide results for wavelengths shorter than 500 nm but analyzed the wavelength dependence of the ocular straylight for angles from 0.5 to 6.0° (Coppens et al. analyzed angles greater than 3.5°). In our work, the blue stimulus used in the C-Quant was centered at 450 nm. However, they observed an increase in straylight at 500 nm for wider angles (6°), both in dark and less pigmented eyes. For the dark-eyed individuals, the straylight values were higher at 500 nm compared with straylight at longer wavelengths (650 nm). These results are in agreement with our work, as we obtained high straylight levels for the blue stimuli compared to the other chromatic stimuli, indicating that the fundus contribution to straylight was lower. In this sense, it should be considered to include eyes with different pigmentation levels in the studies, especially for smaller angles, since in lighter eyes the fundus contribution for long wavelengths is expected to be more important. In addition, Fernandes-Costa et al. reported an increase of straylight and poorer chromatic discrimination thresholds with age, although they indicated a small effect of straylight impact on chromaticity thresholds^49^. In our work, age is not expected to produce a significant impact on visual discrimination or straylight, since the participants were young individuals, within the groups in which these authors indicated that there were no significant changes except for the blue-yellow axis (tritan). Another factor to take into account is pupil size, since the increased pupil diameter observed in the Lmax condition for the blue channel contributes to increased halo perception, which is in line with previous studies^6^. In a similar vein, other authors have shown that pupil miosis reduces some night vision disturbances^29^ and some drugs are being developed to alleviate the symptoms in patients with severe night vision disturbances^50^. In our study, we also observed the increase in halo size for the blue stimulus while maintaining the same luminance level, although somewhat less intensively. In the condition of equal stimulus luminance, no changes in pupil diameter with stimulus chromaticity are expected, as some authors have shown^51^. This indicates that while pupil size has an important effect, there are other important causes that increase the perception of night vision disturbances. Moreover, some authors have shown that straylight has a weak dependence on pupil size (for diameters from 2 to 7 mm), whereby increasing pupil diameter slightly increases the straylight value^52^. However, the authors concluded that straylight values measured under photopic conditions are also valid for mesopic and scotopic conditions.

The direct significant correlation between the achromatic VDI and straylight parameter is similar to that which we have reported in previous studies wherein forward scattering was induced using Bangerter foils and fog filters^10,37^. Using an achromatic stimulus, a direct correlation between the VDI and the objective scattering index (OSI) was found in these studies, but also with the straylight parameter evaluated using white light. This led to subjects reporting a greater perception of halos and other positive dysphotopsias when increasing the forward scattering and straylight. It is important to highlight differences in the way OSI and straylight are measured, given that infrared light is used to objectively measure intraocular scattering through the OSI, while white light is used to measure intraocular straylight^10^. On the contrary, in the present work, we did not find any significant correlation between VDI and straylight for the chromatic conditions, showing a high variability in the results. This indicates that a possible limitation of the study may be that both parameters are dependent on subjective factors, but also the sample size analyzed. On the other hand, it could be studied whether the dark adaptation has any influence on the dispersion of VDI data, considering that the cones are the photoreceptors involved in the task of perceiving the stimuli around the central stimulus (foveal vision). In this regard, further research should be conducted with larger samples and including objective ocular measurements, as ocular aberrations and optical quality of the eye, as well as taking into account different dark adaptations.

Impaired visual discrimination associated with a blue stimulus can also be explained, at least partially, by optical aberrations of the eye. Ocular aberrations should be considered, as they affect the central part of the point spread function (PSF), within a visual angle of 1°, at which visual discrimination capacity was assessed. Some authors have shown a slight increase of higher order aberrations with wavelength using different methods^53,54^ and, however, an increases or decreases in optical quality depending on the metrics used^53^. Other authors have also demonstrated an important effect of longitudinal chromatic aberration, showing little change in higher-order aberrations, which are predominantly due to the primary spherical aberration^55^. In this sense, one study reported an average longitudinal chromatic aberration of 1.82 D over a range of 420 to 660 nm^56^, being 1.26–1.33 D for a range of 450 to 650 mm according to other authors^53^, a range that is better adapted to the chromatic stimuli of the halo. While light is being focused to form images inside the eye, short wavelengths would focus in front of the retina, becoming myopic, thus producing a defocus for these stimuli, and contributing to the perception of halos. In the present study, we performed a subjective refraction with binocular balance and took the lens power that gave the first response with letters of equal clarity on both the red and green sides as the endpoint. Therefore, the greatest defocus was caused by the blue stimulus (myopic defocus), while the green and red stimuli presented myopic and hypermetropic defocus, respectively, of approximately equal magnitudes. Some authors demonstrated a significant effect of defocus on foveal detection thresholds for white circular stimuli of different sizes, especially for hyperopic refractive errors in small simuli^57^. More specifically, a contrast sensitivity of slightly less than 20, threshold contrast of 5%, was found for a myopic defocus of 1.0 D and a stimulus size of 0.2°. In our study, the minimum contrast condition in central and peripheral simuli was for the L_Bmax conditions (Michelson contrasts of 0.975 and 0.957, respectively), which would correspond to much higher contrast levels compared to the contrast thresholds reported by means of contrast sensitivity, although lower contrast sensitivity would be expected for smaller stimuli according to the results of these authors. Differences in VDI have been reported for defocus levels of 0.75 and 1.25 D, but under less favorable conditions, as the monovision conditions were simulated^6^. Detection of the small stimuli around the central stimulus could be also influenced by cone distribution in the retina (Halo test was performed in foveal vision) since only 7% of the cones in the central fovea correspond to S cones (short wavelength sensitive cones)^58^, which contributes to a worse detection of small blue stimuli in foveal vision, an aspect that would be more affected in the Halo test conditions under the influence of the central stimulus of higher luminance. Therefore, the low density of S cones in the fovea and lower contrast thresholds for small stimuli due to defocus, together with a contribution of the Rayleigh pattern due to forward scattering, could explain the increased in halo perception and straylight with blue stimuli, although further research is needed in this regard.

Regarding night myopia, Artal et al. found that it is mainly due to an accommodation shift at low luminance levels, while the effect of chromatic aberrations is quite limited^59^. They demonstrated that myopic shifts were moderate and a defocus of more than 0.50 D was only achieved in abnormally low light conditions, which are uncommon in everyday life. Another study found that straylight is not influenced by dioptric blur^60^, so the effect of night myopia due to defocus is not expected to have a significant impact on our visual discrimination and straylight results. It should be noted that Artal et al. only studied white light and green light at 550 nm, nor did they perform any comparisons using discrimination tasks under night vision conditions^59^. However, the authors indicated the need for studies to assess vision under natural conditions, i.e., binocular vision, as they only assessed visual function monocularly. In our study, we assessed an important aspect of visual performance under night vision conditions, i.e., visual discrimination (halo perception), both monocularly and binocularly with different stimuli using white, red, green, and blue light. Our results show that, under natural viewing conditions (binocularly), the visual discrimination ability improves with respect to monocular results.

Our findings are relevant to night vision scenarios where color information is important, such as environments with streetlights, illuminated signs, LED advertising displays, and vehicle headlights, as many of these light sources have a blue component due to the increasing use of LEDs. Our results could be of special interest in terms of night driving, as the driver’s visual performance is affected by the headlights of oncoming cars. In this regard, the background luminance of the Halo test monitor is within the range of the spatial luminance distribution in the forefield of a vehicle without considering oncoming traffic^61^. The headlights of oncoming cars produce varying lighting conditions, so it is important to assess night vision disturbances under the influence of a bright light source, just as the Halo test does, although with a dimmer light source than car headlights.

The use of LEDs in vehicle headlights has become widespread in recent years^62^. LED headlights have a spectral distribution with a peak in the blue region, typically at a wavelength of ~ 460 nm. Some studies have demonstrated that the light emitted from LED headlamps produces more glare than conventional headlamps, such as those with halogen or discharge bulbs^63^. This is due to a higher luminance and the contribution of shorter wavelengths. At this point we should include the findings of our work: a stronger intraocular straylight and a greater perception of halos at these wavelengths. To minimize the negative effects of glare from vehicle headlights, research is currently underway to develop adaptive high-beam controlling systems in headlamps composed of LED modules^64^ or anti-glare rear view mirrors^65^, among other innovations. However, besides work on possible solutions for lighting systems, it is important to assess how drivers’ night vision is affected by the headlights of oncoming vehicles, either because of glare or the perception of halos and other visual disturbances. These disturbances depend on the visual quality of the observer’s eyes (eye diseases, ocular media, binocular vision, etc.)^13,15,41^. Thus, driving performance could be affected^39,66^, as could the perception of traffic signs or the detection of pedestrians crossing the road or on the sidewalk^67,68^, subsequently increasing the risk of road traffic accidents. Accordingly, more research is needed into driver visual performance while also considering the vehicle lighting system (headlights, taillights, type of headlights, etc.) and not just the driver’s visual condition. The participants in our study were young and had optimal visual performance in terms of visual acuity, visual discrimination, and intraocular straylight. We would also like to highlight that night vision tests for chromaticity should be performed in subjects with different characteristics, at least for achromatic and blue stimuli. It is particularly important to study night vision performance in diseased eyes^13,15^ but also after different refractive surgery techniques (IOL implantation, LASIK, SMILE, etc.), as halo perception and other dysphotopsias are one of the main postoperative complaints of these patients^12,69,70^ and some multifocal IOLs can influence spatio-chromatic vision^71^. There is also a growing research interest in the effect of blue-light filtering intraocular lenses^72,73^ and optimizing new refractive surgery techniques to minimize positive dysphotopsias.

## Conclusions

The results of our study indicate that visual discrimination under low illumination conditions (perception of halos, night vision disturbances) depends on the chromaticity of the stimuli, wherein both the monocular and binocular visual impairment is greater for the blue stimulus. The same trend was observed when the visual stimuli were under equal luminance, but also under conditions of maximum luminance (provided by the test monitor for each stimulus). However, the perception of halos was greater at lower luminance, indicating the important influence of increasing pupil size, but not as significant as the influence of the blue stimulus (which produced the greatest halo perception effect) compared to other stimuli. This visual function was better under binocular viewing conditions for all the stimuli (achromatic, red, green or blue) and luminance levels we tested, underlining the superiority of binocular compared to monocular vision. We observed an important increase in intraocular straylight for blue light, demonstrating a major contribution of shorter wavelengths, thus resulting in a stronger luminous veil over the retina and, therefore, more glare. We also found that the visual disturbance index correlated positively with intraocular straylight in the achromatic condition, in such a way that as intraocular straylight increases, visual discrimination declines and, therefore, visual halos become more prominent. While these results are of interest in everyday activities, such as the mobility of pedestrians in the city at night, where there are various chromatic visual stimuli and intense light sources, our findings could be of greater relevance to driving and the automotive sector, as driver vision may be affected by the type of headlights of oncoming cars which are increasingly incorporating the use of LED modules. Our results may also be of interest to drivers who have undergone some type of refractive surgery or who have some type of ocular pathology and that it is important to evaluate night visual symptoms both with white light and taking into account some stimuli with a specific chromaticity.

## Data Availability

The data that support the findings of this study are available from the corresponding author upon reasonable request.

## References

[1] Bourne RRA (2021). Trends in prevalence of blindness and distance and near vision impairment over 30 years: An analysis for the global burden of disease study. Lancet Glob. Health.

[2] McLeod SD (2001). Beyond Snellen acuity—The assessment of visual function after refractive surgery. Arch. Ophthalmol..

[3] Piano MEF, O'Connor AR (2013). The effect of degrading binocular single vision on fine visuomotor skill task performance. Investig. Ophthalmol. Vis. Sci..

[4] Datta S (2008). The importance of acuity, stereopsis, and contrast sensitivity for health-related quality of life in elderly women with cataracts. Investig. Ophthalmol. Vis. Sci..

[5] Rosen ES (2005). Night vision disturbance. J. Cataract Refract. Surg..

[6] Castro JJ, Soler M, Ortiz C, Jimenez JR, Anera RG (2016). Binocular summation and visual function with induced anisocoria and monovision. Biomed. Opt. Express.

[7] Helgesen A, Hjortdal J, Ehlers N (2004). Pupil size and night vision disturbances after LASIK for myopia. Acta Ophthalmol. Scand..

[8] Puell MC, Perez-Carrasco MJ, Barrio A, Antona B, Palomo-Alvarez C (2013). Normal values for the size of a halo produced by a glare source. J. Refract. Surg..

[9] Yao L (2020). Relationships between haloes and objective visual quality in healthy eyes. Transl. Vis. Sci. Technol..

[10] Castro-Torres JJ, Martino F, Casares-Lopez M, Ortiz-Peregrina S, Ortiz C (2021). Visual performance after the deterioration of retinal image quality: Induced forward scattering using Bangerter foils and fog filters. Biomed. Opt. Express.

[11] Bidgoli S, Alió JL, Alio JL, Azar DT (2018). Management of Complications in Refractive Surgery.

[12] Kohnen T, Suryakumar R (2021). Measures of visual disturbance in patients receiving extended depth-of-focus or trifocal intraocular lenses. J. Cataract Refract. Surg..

[13] Ortiz C, Castro JJ, Alarcon A, Soler M, Anera RG (2013). Quantifying age-related differences in visual-discrimination capacity: Drivers with and without visual impairment. Appl. Ergon..

[14] Babizhayev, M. A. Disability glare effects and cataract as a problem of road safety in driving. In *Cataracts: Causes, Symptoms, and Surgery* 1–36 (2010).

[15] Castro JJ, Jimenez JR, Ortiz C, Alarcon A, Anera RG (2011). New testing software for quantifying discrimination capacity in subjects with ocular pathologies. J. Biomed. Opt..

[16] Klyce SD (2007). Night vision disturbances after refractive surgery: Haloes are not just for angels. Br. J. Ophthalmol..

[17] Palomo-Alvarez C, Puell MC (2015). Capacity of straylight and disk halo size to diagnose cataract. J. Cataract Refract. Surg..

[18] Schwiegerling J (2006). Recent developments in pseudophakic dysphotopsia. Curr. Opin. Ophthalmol..

[19] Puell MC, Palomo-Alvarez C (2017). Effects of light scatter and blur on low-contrast vision and disk halo size. Optom. Vis. Sci..

[20] Ferreira-Neves H (2015). Validation of a method to measure light distortion surrounding a source of glare. J. Biomed. Opt..

[21] Buckhurst PJ (2015). Tablet App halometer for the assessment of dysphotopsia. J. Cataract Refract. Surg..

[22] Ruiz-Mesa R, Abengozar-Vela A, Aramburu A, Ruiz-Santos M (2017). Comparison of visual outcomes after bilateral implantation of extended range of vision and trifocal intraocular lenses. Eur. J. Ophthalmol..

[23] Savini G (2019). Functional assessment of a new extended depth-of-focus intraocular lens. Eye.

[24] Gutierrez R, Jimenez JR, Villa C, Valverde JA, Anera RC (2003). Simple device for quantifying the influence of halos after lasik surgery. J. Biomed. Opt..

[25] Anera RG, Castro JJ, Jimenez JR, Villa C, Alarcon A (2011). Optical quality and visual discrimination capacity after myopic LASIK with a standard and aspheric ablation profile. J. Refract. Surg..

[26] Zhao WX (2023). Evaluating early-stage disk halo changes after small incision lenticule extraction. Eur. J. Ophthalmol..

[27] Hastings GD, Marsack JD, Thibos LN, Applegate RA (2018). Normative best-corrected values of the visual image quality metric VSX as a function of age and pupil size. J. Opt. Soc. Am. Opt. Image Sci. Vis..

[28] Wang Y, Zhao KX, Jin Y, Niu YF, Zuo T (2003). Changes of higher order aberration with various pupil sizes in the myopic eye. J. Refract. Surg..

[29] Xu RF, Kollbaum P, Thibos L, Lopez-Gil N, Bradley A (2018). Reducing starbursts in highly aberrated eyes with pupil miosis. Ophthal. Physiol. Opt..

[30] Theeuwes J, Alferdinck J, Perel M (2002). Relation between glare and driving performance. Hum. Factors.

[31] Coppens JE, Franssen L, van den Berg T (2006). Wavelength dependence of intraocular straylight. Exp. Eye Res..

[32] Ginis HS, Perez GM, Bueno JM, Pennos A, Artal P (2013). Wavelength dependence of the ocular straylight. Investig. Ophthalmol. Vis. Sci..

[33] Momeni-Moghaddam H, Goss DA (2014). Comparison of four different binocular balancing techniques. Clin. Exp. Optom..

[34] Puell MC, Perez-Carrasco MJ, Palomo-Alvarez C, Antona B, Barrio A (2014). Relationship between halo size and forward light scatter. Br. J. Ophthalmol..

[35] Macedo-de-Araujo R, Ferreira-Neves H, Rico-del-Viejo L, Peixoto-de-Matos SC, Gonzalez-Meljome JM (2016). Light distortion and spherical aberration for the accommodating and nonaccommodating eye. J. Biomed. Opt..

[36] Cedrun-Sanchez JE (2016). Visual discrimination increase by yellow filters in retinitis pigmentosa. Optom. Vis. Sci..

[37] Martino F (2022). Effect of interocular differences on binocular visual performance after inducing forward scattering. Ophthal. Physiol. Opt..

[38] Castro JJ, Pozo AM, Rubino M, Anera RG, del Barco LJ (2014). Retinal-image quality and night-vision performance after alcohol consumption. J. Ophthalmol..

[39] Casares-Lopez M (2020). Contrast sensitivity and retinal straylight after alcohol consumption: Effects on driving performance. Sci. Rep..

[40] Castro, J. J., Pozo, A. M. & Rubino, M. Color dependence with horizontal-viewing angle and colorimetric characterization of two displays using different backlighting. In *Proc. SPIE 8785, 8th Iberoamerican Optics Meeting and 11th Latin American Meeting on Optics, Lasers, and Applications* 87854C. 10.1117/12.2026171 (2013).

[41] Van den Berg T (2007). Straylight effects with aging and lens extraction. Am. J. Ophthalmol..

[42] van den Berg T, Franssen L, Coppens JE (2009). Straylight in the human eye: Testing objectivity and optical character of the psychophysical measurement. Ophthal. Physiol. Opt..

[43] Labuz G, Reus NJ, van den Berg T (2015). Ocular straylight in the normal pseudophakic eye. J. Cataract Refract. Surg..

[44] Labuz G, Lopez-Gil N, van den Berg T, Vargas-Martin F (2017). Ocular straylight with different multifocal contact lenses. Optom. Vis. Sci..

[45] Cervino A, Montes-Mico R, Hosking SL (2008). Performance of the compensation comparison method for retinal straylight measurement: Effect of patient’s age on repeatability. Br. J. Ophthalmol..

[46] Iijima A, Shimizu K, Kobashi H, Saito A, Kamiya K (2015). Repeatability, reproducibility, and comparability of subjective and objective measurements of intraocular forward scattering in healthy subjects. Biomed. Res. Int..

[47] Martinez-Roda JA (2016). Double-pass technique and compensation-comparison method in eyes with cataract. J. Cataract Refract. Surg..

[48] Stockman A, Sharpe LT (1998). Human cone spectral sensitivities: A progress report. Vis. Res..

[49] Costa MF, Rego LS, Henriques LD, Gaddi CM, Souza GS (2023). Reduced eye optical quality contributes to worse chromatic thresholds in aging. Front. Integr. Neurosci..

[50] Pepose J (2022). A randomized phase 2 clinical trial of phentolamine mesylate eye drops in patients with severe night vision disturbances. BMC Ophthalmol..

[51] Adrian W (2003). Spectral sensitivity of the pupillary system. Clin. Exp. Optom..

[52] Franssen L, Tabernero J, Coppens JE, van den Berg T (2007). Pupil size and retinal straylight in the normal eye. Investig. Ophthalmol. Vis. Sci..

[53] Marcos S, Burns SA, Moreno-Barriusop E, Navarro R (1999). A new approach to the study of ocular chromatic aberrations. Vis. Res..

[54] Manzanera S, Canovas C, Prieto PM, Artal P (2008). A wavelength tunable wavefront sensor for the human eye. Opt. Express.

[55] Liu M, Wang Z-Q, Wang Y, Zuo T, Wang Y (2008). The study of wavelength-dependent wavefront aberrations based on individual eye model. Optik.

[56] Howarth PA, Bradley A (1986). The longitudinal chromatic aberration of the human-eye, and its correction. Vis. Res..

[57] Anderson RS, McDowell RD, Ennis FA (2001). Effect of localized defocus on detection thresholds for different sized targets in the fovea and periphery. Acta Ophthalmol. Scand..

[58] Curcio CA (1991). Distribution and morphology of human cone photoreceptors stained with anti-blue opsin. J. Compar. Neurol..

[59] Artal P, Schwarz C, Canovas C, Mira-Agudelo A (2012). Night myopia studied with an adaptive optics visual analyzer. PLoS ONE.

[60] van den Berg T (2017). The (lack of) relation between straylight and visual acuity. Two domains of the point-spread-function. Ophthal. Physiol. Opt..

[61] Erkan A (2021). Determination of speed-dependent roadway luminance for an adequate feeling of safety at nighttime driving. Vehicles.

[62] Long XM (2015). A review on light-emitting diode based automotive headlamps. Renew. Sustain. Energy Rev..

[63] Kang B, Yong B, Park K (2010). Performance evaluations of LED headlamps. Int. J. Autom. Technol..

[64] Luo CM, Xin GF, Xu H, Tang W (2021). Glare-free high-beam control for oncoming vehicle safety in nighttime. IEEE Consum. Electron. Mag..

[65] Caruso D (2015). Market evaluation, performance modelling and materials solution addressing short wavelength discomfort glare in rear view automotive mirrors. Transl. Mater. Res..

[66] Ortiz-Peregrina S (2020). Intraocular scattering as a predictor of driving performance in older adults with cataracts. PLoS ONE.

[67] Tyrrell RA, Wood JM, Owens DA, Borzendowski SW, Sewall AS (2016). The conspicuity of pedestrians at night: A review. Clin. Exp. Optom..

[68] Hwang AD, Tuccar-Burak M, Goldstein R, Peli E (2018). Impact of oncoming headlight glare with cataracts: A pilot study. Front. Psychol..

[69] Liu XM, Wu XM, Huang YS (2022). Laboratory evaluation of halos and through-focus performance of three different multifocal intraocular lenses. J. Refract. Surg..

[70] Hui N (2022). Comparative analysis of visual quality between unilateral implantation of a trifocal intraocular lens and a rotationally asymmetric refractive multifocal intraocular lens. Int. J. Ophthalmol..

[71] Millan MS, Clave L, Torrents A, Armengol J, Vega F (2023). Spatio-chromatic vision with multifocal diffractive intraocular lens. Eye Vis..

[72] Henderson BA, Grimes KJ (2010). Blue-blocking IOLs: A complete review of the literature. Surv. Ophthalmol..

[73] Cueto AFV (2022). Protector role of intraocular lenses under artificial light conditions. Ophthal. Res..

